# Comparison of the efficacy of 0.5% isobaric bupivacaine, 0.5% levobupivacaine, and 0.5% hyperbaric bupivacaine for spinal anesthesia in lower limb surgeries

**DOI:** 10.1038/s41598-023-29711-9

**Published:** 2023-02-15

**Authors:** Valery Piacherski, Lidziya Muzyka

**Affiliations:** Department of Anesthesiology and Intensive Care, Mogilev Regional Clinical Hospital, A. Kuleshov Str., 3-36, 212026 Mogilev, Belarus

**Keywords:** Outcomes research, Clinical trial design

## Abstract

Various advantages of isobaric bupivacaine, levobupivacaine, and hyperbaric bupivacaine are described. There are no studies reliably determining the benefits of these forms of bupivacaine. The purpose of the study was to compare the efficacy of spinal anesthesia (SA) performed with 0.5% isobaric bupivacaine, 0.5% levobupivacaine, and 0.5% hyperbaric bupivacaine. The clinical study was approved by the ethics committee. The sample size was calculated for a confidence level of 99%. 111 patients were randomly allocated into 3 equal groups for spinal anesthesia in lower limb surgeries. In group 1 (1B) spinal anesthesia was performed with 3 ml of 0.5% isobaric bupivacaine (n = 37); in group 2 (2L)—3 ml of 0.5% levobupivacaine (n = 37), in group 3 (3H)—3 ml of 0.5% hyperbaric bupivacaine (n = 37). The criterion for assessing the effectiveness of anesthesia was the need to switch to another type of anesthesia (criterion-no anesthesia), or the need for additional use of narcotic analgesics or local anesthesia during surgery (criterion-reporting of painful feelings during the operation). In 1B anesthesia efficiency by the criterion of additional intraoperative analgesia was 100% (37 patients; 95% CI [0.88–1.0]); 2L—86.4%; (31 patients; 95% CI [0.68–0.92]); 3H—72.9% (27 patients; 95% CI [0.56–0.84]). There were significant differences between groups 1B and 2L: p < 0.05 (p = 0.0104). There were no significant differences between groups 2L and 3H (p = 0.2587). All patients in group 1B developed complete sensory block (++) within 4 (3; 5) min. In group 2L complete sensory block developed in 34 patients (89.4%) within 9 (5; 14) min, in group 3H sensory block developed in all patients within 3 (2.5; 4). The duration of analgesia period between 1B and 2L did not statistically differ (p = 0.73). In 3H the duration of analgesia was 170 (150; 200) min. The study found 83.7% efficacy of levobupivacaine and 72.9% efficacy of hyperbaric bupivacaine in comparison with isobaric bupivacaine (100%) when administered intrathecally in equal volumes and amounts (by the criterion of additional intraoperative analgesia).

Trial registration: NCT05184465 (Initial Release: 12/01/2021; date of first publication—11/01/2022).

## Introduction

More than a hundred years ago, a short article was published on the anesthesiologist's choice among not only the various local anesthetics for performing spinal anesthesia, but also on the choice between isobaric and hyperbaric forms of local anesthetic^1^. It would seem that in a hundred years the question of choosing the form of a local anesthetic for an anesthesiologist should be removed. However, life and everyday professional life leave open the question of well-known (at first glance) local anesthetics.

About 15 million spinal anesthesia procedures are performed worldwide each year^2^. In the daily practice of the anesthesiologist for intrathecal use there are various local anesthetics such as bupivacaine, hyperbaric solution of bupivacaine, ropivacaine and levobupivacaine. From 1946 to 2017, only 16 studies comparing the clinical efficacy of isobaric and hyperbaric bupivacaine in nonpregnant patients have been conducted according to various databases^2^. The small sample size and high heterogeneity of these results suggest that all results should be treated with caution. And, there is no conclusive evidence in favor of isobaric or hyperbaric bupivacaine regarding efficacy or side effects in the general surgical population^2^.

The literature describes such advantages of levobupivacaine as less cardiotoxicity, longer period of analgesia, more pronounced activity against sensory fibers than against motor fibers^3^. In some studies it has been shown that levobupivacaine is equal to isobaric bupivacaine in efficacy^4,5^. The efficacy of hyperbaric levobupivacaine equivalent to hyperbaric bupivacaine when administered intrathecally has also been shown on volunteers^6^.

However, in the literature there are different data on clinical efficacy of levobupivacaine in comparison with ropivacaine and levobupivacaine. So during operations on extremities out of 20 patients surgical anesthesia developed in 18 patients^7^. Fattorini et al. in their study stated the same effectiveness of bupivacaine and levobupivacaine, but when using levobupivacaine in one patient general anesthesia was used due to insufficient spinal anesthesia^8^. Other studies also reported similar efficacy of the two drugs, but surgical satisfaction with intraoperative anesthesia was 92.9% for bupivacaine and 83.9% for levobupivacaine for knee arthroscopy^4^.

In their study, Gautier et al. noted significantly lower efficacy of levobupivacaine in caesarean section compared to bupivacaine and ropivacaine for intrathecal use: 80% vs. 90% and 87%, respectively^9^.

According to Heng Sia et al. there is no clear evidence of the advantage of hyperbaric bupivacaine over isobaric bupivacaine for spinal anesthesia for cesarean section^10^. The authors also noted that adequate randomized clinical trials with clear definitions, criteria and methodology for evaluating the transition to general anesthesia, requirements for additional analgesia, nausea, vomiting and sensory testing are needed^10^.

There is no clear practical guide to help anesthesiologists make informed decisions about the use of some form of intrathecal bupivacaine in non-cesarean surgery. Carefully designed, adequately conducted studies can provide further results that will contribute to sound clinical decision making^2^.

Given the above, the aim of the study is to compare the effectiveness of spinal anesthesia (SA) performed with 0.5% isobaric bupivacaine solution, 0.5% levobupivacaine solution and 0.5% hyperbaric bupivacaine solution in equivalent volumes in lower limb surgeries.

## Materials and methods

The clinical double blind randomized study was approved by the ethics committee of our institution (protocol dated 01/28/2017). All methods were performed in accordance with the relevant guidelines and standards. All experimental protocols were approved by the Ethics Committee of the Mogilev Regional Clinical Hospital. The study is registered on ClinicalTrials.gov—Identifier: NCT05184465 (Initial Release: 12/01/2021; date of first publication—11/01/2022).

**Figure 1:**
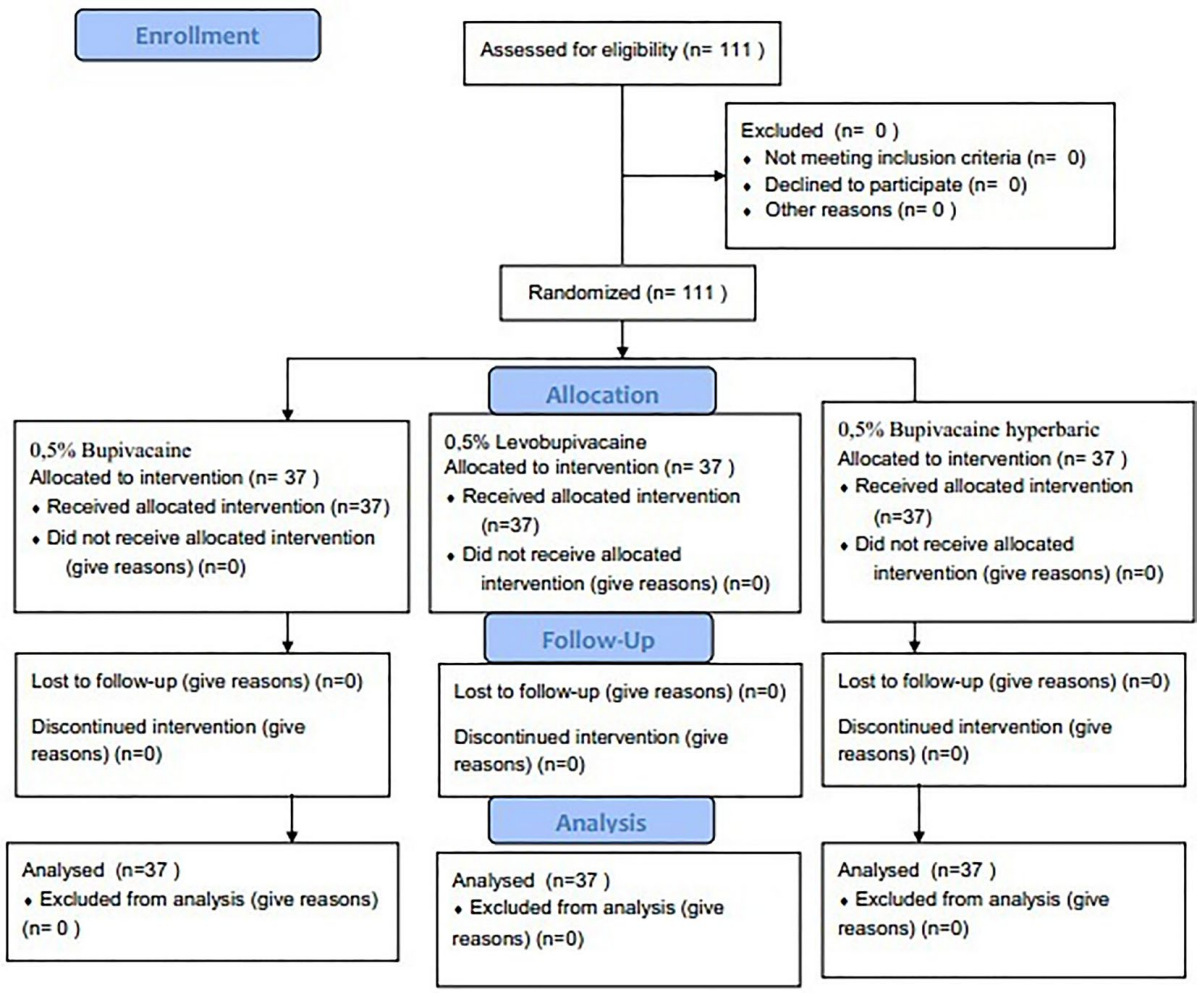
Flow chart on patient distribution.

111 patients who underwent surgical intervention on the hip, thigh, and knee joints were included in the study (Fig. 1).

Criteria for inclusion of patients in the study: indications for surgical intervention on the hip, thigh, and knee joints, the need for anesthetic support (spinal anesthesia).

Exclusion criteria: patient refusal to use the proposed type of anesthesia, age < 18 years, body mass index not > 39, physical status according to ASA > 3, history of allergic reactions to the drugs used, coagulopathy, infectious skin lesions in the injection area, neurological or neuromuscular diseases, severe liver disease or renal failure, inability to cooperate with the patient.

A written informed consent was obtained from all patients or their legal guardians to participate in the clinical trial. And we obtained written informed consent from all patients or their legal guardians about the type of anesthesia and possible complications of regional anesthesia. After obtaining informed consent, patients were randomized into groups using a random number generator (numbers in envelopes). The random assignment sequence was created by an anesthesiologist not involved in the study. Randomization: sealed envelopes with a type of anesthesia.

Spinal anesthesia was administered to the patients for anesthetic support of the surgical intervention. Patients were randomly allocated into three groups: in group 1 (1B) spinal anesthesia was performed with 3 ml of 0.5% bupivacaine (n = 37); in group 2 (2L) spinal anesthesia was performed with 3 ml of 0.5% levobupivacaine (n = 37); in group 3 (3H) 3 ml of 0.5% hyperbaric bupivacaine solution (n = 37) was used for subarachnoid injection. The anesthesiologist, who was not involved in the study, prepared the anesthetic solution immediately before the injection.

Intrathecal injections were performed with a 24G or 25G «Pencil point» needle in the L3–L4 interval. Spinal puncture was performed while the patient was sitting on the table. Then the patient was placed on his back. The patient was positioned for surgery 30 min later.

Solutions for spinal anesthesia were prepared by an anesthesiologist who was not involved in the anesthesia. Spinal anesthesia was performed by an anesthesiologist with many years of experience, who performs 10–15 spinal anesthesias per week.

The peripheral vein was catheterized on the operating table before anesthesia. SPO_2_, ECG, HR, thermometry, and nBP were monitored during anesthesia and surgery.

Surgery was allowed to start after 40 min if the upper level of the sensory block reached the Th10 segment.

The criterion for assessing the effectiveness of anesthesia was the need to switch to another type of anesthesia (criterion-no anesthesia), or the need for additional use of narcotic analgesics or local anesthesia during surgery (criterion-reporting of painful feelings during the operation). Criteria for the use of additional anesthesia: the patient's complaint of pain in the surgical area.

The block was evaluated by an anesthesiologist who was not involved in the study. Sensory block quality was recorded on both sides along the midclavicular line, assessing changes in needle prick sensation. Skin sensitivity was assessed every 2 min for 40 min. The following scale was used to assess sensory block, where: “++”—complete sensory block (anesthesia); “+”—not complete sensory block, the patient could not differentiate the type of stimulus; “−”—skin sensitivity preserved to the full extent.

The development of motor block was assessed using the Bromage scale (0–3) for 40 min. End of motor block was defined as the appearance of the first movements in the lower extremities.

The duration of postoperative analgesia was assessed by interviewing the patient in the postoperative period. The duration of analgesia was assessed in the postoperative period every 30 min. Pain sensations were assessed by visual analog scale (VAS) from 0 cm (no pain) to 10 cm (unbearable pain). The end of analgesia was considered the moment when the patient noted the onset of pain (1–2 points). If painful sensations appeared in the postoperative wound area (1–2 points), we injected intramuscularly 2%-1 ml of Promedol for postoperative analgesia. The duration of analgesia was assessed by an independent anesthesiologist, who was not involved in the study.

For the additional intraoperative analgesia criterion, the null hypothesis implies that the degree of success in the compared groups (for each local anesthetic) is the same (p > 0.05). If the null hypothesis should be rejected after a statistical test (p < 0.05), it is concluded that one of the groups is superior to the others on this indicator. The sample size was calculated for a confidence level of 99% and statistical power of 99% and a type 1 error of 0.01 (taking into account the effectiveness of performing spinal anesthesia with isobaric bupivacaine 0.5% in orthopedics at our institution). Considering previous studies in this area, where the sample size ranged from 29 to 40 patients^3,4^, we included an additional 30 patients in each group to increase validity (total number of patients in each group 37).

Statistical processing of the obtained data was performed using Statistica 7.0 software. The data were presented as median and quartiles (25th% and 75th%) and also as mean and standard deviation. Differences between the groups were considered statistically significant at p < 0.05. The primary endpoint was the need to switch to another type of anesthesia or the need for additional narcotic analgesics or local anesthesia either initially or during surgery. The frequencies of the binary feature in the two unrelated (independent) groups were compared by analysis of a contingency table (2 × 2). Classic Pearson's χ^2^ test (Chi-square) was used; Fisher exact p test was applied in the presence of values of the phenomena of 5 and less. Differences between the groups were considered statistically significant at p < 0.05. 95% CI for categorical data calculated using Wald's method.

Secondary endpoints: time of sensory and motor block development, duration of postoperative analgesia and motor block. The groups were compared using nonparametric Mann–Whitney test. Differences between the groups were considered statistically significant at p < 0.05.

## Results

The study was performed from 12/18/2017 to 12/13/2018. The patient groups had no statistically significant differences in age and body weight. Characteristics of the studied groups of patients are presented in Table 1. All anesthesia was performed without complications.

**Table 1:** Characteristics of the study groups of patients.

Characteristics of groups	Group 1B n = 37	Group 2L n = 37	Group 3H n = 37
Age, years	59 (55; 63)	49 (36; 63)	57 (53; 61)
Gender (m/f)	15/22	24/13	20/17
Body weight, kg	84 (76; 94)	80 (70; 90)	80 (70; 88)
Height, cm	170 (164; 174)	168 (162; 174)	170 (164; 175)
Operation on the femur (osteosynthesis, removal of osteosynthesis)	3	6	8
Total hip arthroplasty	21	14	29
Total knee arthroplasty	9	2	0
Arthroscopy of the knee	2	12	0
Osteosynthesis of tibia and fibula	1	3	0
Removal of metal structure from the pelvis	1	0	0
Duration of surgery, min	115 (85; 130)	100 (55; 130)	120 (115; 130)

Values are median (1Q, 3Q).

Group 1B—0.5% bupivacaine, Group 2L—0.5% levobupivacaine, Group 3H—0.5% hyperbaric bupivacaine.

Gender (m/f)—male/female.

None of the patients in group 1B had the need to switch to another type of anesthesia or the need for additional use of narcotic analgesics or local anesthesia during surgery (Table 2). In 6 patients (16.2%) of Group 2L, there was a need to control pain syndrome intraoperatively using narcotic analgesics (fentanyl) or local anesthesia during surgery. There were significant differences between the groups in the need for additional intraoperative pain relief: χ^2^ = 6.53, at p = 0.0104; Fisher exact p test = 0.0125. Thus, when levobupivacaine was used for spinal anesthesia, no additional intraoperative anesthesia was required in 31 patients (83.7%).

**Table 2:** Characteristics of spinal anesthesia in the study groups.

Evaluated parameters	Isobaric bupivacaine (1B)	Levobupivacaine (2L)	Hyperbaric bupivacaine (3H)
Without additional intraoperative anesthesia (patients, %)	37 (100%)*^†^	31 (83.7%)*	27 (72.9%)^†^
Complete sensory block (patients, %)	37 (100%)	33 (89.1%)	37 (100%)
Complete motor block (patients, %)	37 (100%)*	32 (86.4%)*^‡^	37 (100%)^‡^
Time to develop a complete sensory block (min)	4 (3; 5)*	10 (5; 14)*^‡^	3 (2.5; 4)^‡^
Time of development of the full motor block (min)	8 (6; 11)*	15 (10; 22.5)*^‡^	4 (2.5; 5)^‡^
Duration of analgesia (min)	241 (212; 266)^†^	245 (201.5; 288.5)^‡^	170 (132; 202)^†‡^
Duration of motor block (min)	217 (199; 255)^†^	235 (199; 275)^‡^	177 (150; 200)^†‡^

Values are median (1Q, 3Q).

Symbols: * ^†^^‡^indicators marked with the same symbols have significant differences between each other in the horizontal line on this indicator (p < 0.05).

All patients in group 1B developed a complete sensory block (++) within 4 (3; 5) min. In group 2L full sensory block developed in 33 patients (89.1%) within 10 (5; 14) min, statistically significant differences were obtained between the groups in the rate of sensory block development, p = 0.000. No significant differences were obtained in the quality of sensory block development: in the bupivacaine group, sensory block developed in 100% of patients in the levobupivacaine group in 89.1%: χ^2^ = 4.23, at p = 0.0398; however Fisher exact p = 0.0574.

Complete motor block (Bromage 3) developed in all group 1B patients within 8 (6; 11) min. In group 2L, a complete motor block developed in 32 patients (86.4%) within 15 (10; 22.5) min. Significant differences between the groups were obtained in terms of time of motor block development, p = 0.00005. Significant differences were obtained in the quality of motor block development: in the bupivacaine group motor block developed in 100% of patients, in the levobupivacaine group in 86.4%: χ^2^ = 5.36, at p = 0.0206; Fisher exact p = 0.0271.

The duration of analgesia period did not statistically differ between the groups and was 241 (212; 266) min in Group 1B and 245 (201.5; 288.5) min in Group 2L, p = 0.73.

The duration of motor block in group 1B was 217 (199; 255) min, in group 2L—235 (199; 275) min. No significant differences were obtained, p = 0.28.

In 10 patients (27.02%) in group 3H there was a need for pain control intraoperatively using narcotic analgesics (fentanyl) or local anesthesia. There were significant differences between groups 1B and 3H in the need for additional intraoperative analgesia: χ^2^ = 11.56, at p = 0.0007; Fisher exact p = 0.0005. Thus, when using hyperbaric bupivacaine for spinal anesthesia, no additional intraoperative analgesia was required in 27 patients (72.9%) of 37.

There was no significant difference between groups 2L and 3H in the need for additional intraoperative analgesia: χ^2^ = 1.28, at p = 0.2587; Fisher exact p = 0.1988.

Complete sensory and motor block (Bromage 3) in group 3H developed in 100% of cases. The time for development of complete sensory block was 3 (2.5; 4) min. There were no significant differences between groups 1B and 3H in time to develop complete sensory block (p = 0.21). When hyperbaric bupivacaine was used, sensory block developed significantly faster than when levobupivacaine was used (15 (10; 22.5) min): p = 0.000.

The time of development of complete motor block in group 3H was 4 (2.5; 5) min. There were no significant differences between groups 1B and 3H in the time of development of complete motor block (p = 0.22). There were significant differences between groups 2L (15 (10; 22.5) min) and 3H (p = 0.000) for time of motor block development.

The duration of motor block in group 3H was 177 (150; 200) min. This was on average 58 min shorter than with levobupivacaine and 64 min shorter than with isobaric bupivacaine. Significant differences were obtained between groups 3H and 1B, 2L (p = 0.000006 and p = 0.000029 respectively).

The duration of analgesia period with hyperbaric solution (3H) was 170 (132; 202) min, which was on average 75 min less than with levobupivacaine and 71 min less than with isobaric bupivacaine. Significant differences were obtained between groups 3H and 1B, 2L, p < 0.05 (p = 0.000007 and p = 0.000007, respectively).

The results of the study with a 95% confidence interval are presented in Table 3.

**Table 3:** Characteristics of spinal anesthesia in the study groups with 95% CI.

Evaluated parameters	Isobaric bupivacaine (1B) n = 37 Mean (SD) [lower and upper 95% CI]	Levobupivacaine(2L) n = 37 Mean (SD) [lower and upper 95% CI]	Hyperbaric bupivacaine (3H) n = 37 Mean (SD) [lower and upper 95% CI]
Without additional intraoperative anesthesia (patients)	37 [0.88–1.0]	31 [0.68–0.92]	27 [0.56–0.84]
Complete sensory block (patients)	37 [0.88–1.0]	33 [0.74–0.96]	37 [0.88–1.0]
Complete motor block (patients)	37 [0.88–1.0]	32 [0.71–0.94]	37 [0.88–1.0]
Time to develop a complete sensory block (min)	4.72 (3.1) [3.68–5.75]	9.72 (5.01) [8.04–11.39]	3.58 (1.85) [2.96–4.19]
Time of development of the full motor block (min)	9.35 (4.86) [7.78–10.9]	17.12 (8.89) [14.15–20.08]	4.66 (3.51) [3.48–5.83]
Duration of analgesia (min)	245.59 (47.64) [229.7–261.4]	248.61 (80.53) [221.76–261.4]	173.89 (66.93) [151.57–196.2]
Duration of motor block (min)	228.72 (55.05) [210.3–247]	242 (67.02) [219.65–264.34]	180.05 (47.68) [164.15–195.94]

*CI* confidence interval, *SD* standard deviation.

Characteristics of cases when an additional type of anesthesia was required.

Levobupivacaine (6 cases): at the beginning of the operation—3 cases (1st case—fentanyl; 2nd case (for skin incision)—local anesthesia + propofol; 3rd case (sutures to the skin)—local anesthesia. End of the operation (sutures on the skin)—local anesthesia. Beginning and end of the operation (for skin incision and skin suture)—1st case—local anesthesia, 2nd case—fentanyl intravenously.

Bupivacaine hyperbaric (10 cases): middle of the operation—1st case—endotracheal anesthesia; at the end of the operation (8 cases)—local anesthesia-1, fentanyl-5, fentanyl + propofol—2; middle and end of the operation—1 case—fentanyl intravenously.

Nausea and vomiting were not observed in any patient during the intraoperative period. All surgical interventions in each group were completed successfully, there were no complications.

## Discussion

Our data on the need for additional anesthesia during surgery (84.2% efficiency) are comparable to those obtained by Gautier et al. in the subarachnoid use of levobupivacaine in caesarean section, where its efficiency was 80%^9^. Similar data were obtained by del-Rio-Vellosillo et al. who reported that surgical satisfaction with intraoperative analgesia was 92.9% for bupivacaine and 83.9% for levobupivacaine for anesthesia during knee arthroscopy^3,6^. It should be noted that we increased the amount of levobupivacaine and bupivacaine from 12.5 to 15 mg in comparison with the del-Rio-Vellosillo et al. study, but the effectiveness of levobupivacaine was not increased relative to this study.

However, other studies have shown high efficacy of levobupivacaine in subarachnoid use^6,10^, but in the study of Gautier et al. 2 patients out of 20 required additional anesthesia during surgery due to ineffective subarachnoid anesthesia^9^ when using levobupivacaine. Also in the study of Glaser et al. there was one unsuccessful spinal anesthesia, which was defined as a technical defect^4^.

Time of sensory block development in our study was statistically significantly faster in bupivacaine group than in levobupivacaine group (4 (3; 5) min vs. 9 (5; 14) min), which is consistent with the data obtained in other studies^3,9^. But in the study of Fattorini et al. and Singh, there was no difference in the development of sensory block^7,10^. Thus, the existing data on the difference in the rate of development of sensory block are contradictory. In these studies and in our study, there was no dependence of the rate of block development on the amount of bupivacaine and levobupivacaine.

Efficiency of development of complete motor block in levobupivacaine group was 86.8%, and significantly different from bupivacaine group. These data are consistent with previous studies^9,11^, despite the fact that in our study slightly higher amount of levobupivacaine was used. But according to other authors, levobupivacaine was not inferior to bupivacaine in quality of motor block^3,7,10^.

In our study we obtained no significant differences in the duration of postoperative analgesia. These results are similar to the results of other studies^9,11^ However, according to other authors, the duration of anesthesia is still longer when bupivacaine is used^10^.

The authors of a major review found little evidence for the need to switch to general anesthesia and adjuvant analgesia between hyperbaric or isobaric bupivacaine groups. This is due to the rarity of these results, variability in doses, use of adjuvant drugs, and differences in regional anesthesia techniques. Any possible, in the authors' opinion, benefits of hyperbaric bupivacaine should be confirmed in larger randomized trials. In future studies, the criteria for switching to general anesthesia should be defined objectively and applied uniformly^11^. It should be noted that in our study there were significant differences between isobaric bupivacaine and hyperbaric bupivacaine groups regarding the need for additional intraoperative analgesia (we used equal concentrations and volumes of local anesthetic, observed the same level of intrathecal administration, the criterion for additional intraoperative analgesia was the patient's statement of pain during surgery).

According to Heng Sia et al. there is no clear evidence of superiority of hyperbaric anesthesia over conventional bupivacaine for spinal anesthesia in cesarean section^10^. Our data show the superiority of isobaric bupivacaine over hyperbaric in some parameters, including additional intraoperative analgesia, but they are obtained in operations on the lower extremities.

At the same time, a study published in 1984 showed fewer failures when using hyperbaric solution than isobaric solution^12^. In contrast, our study showed a significant advantage of isobaric bupivacaine.

According to other authors, levobupivacaine was not inferior to bupivacaine in quality of motor block, but anesthesia duration was longer when bupivacaine was used^13^.

In Huang et al. study, it was noted that when using hyperbaric bupivacaine 0.5% intrathecally, about 70% of sensory block variants can be predicted. And about 30% are incomprehensible variants and they require study^14^. It can be assumed that such unexplained variations may be in relation to sensory, motor block, pain sensitivity (for various forms of bupivacaine: hyperbaric form and levobupivacaine). And also, perhaps these variants can occur at various stages of the perioperative period. All this needs more research.

The limitations of the study are that we did not compare the hemodynamic effects of the three drugs, nor did we evaluate study groups for the development of postoperative nausea and vomiting.

In conclusion, our study determined 83.7% efficacy of levobupivacaine and 72.9% efficacy of hyperbaric bupivacaine compared with isobaric bupivacaine (100%) when administered intrathecally in equal volumes and amounts (according to the criteria of additional intraoperative analgesia). The slowest development of sensory and motor block was noted in levobupivacaine. The longest postoperative analgesia was observed for isobaric bupivacaine and levobupivacaine. It should be noted that the data of different authors on the efficacy of levobupivacaine and hyperbaric bupivacaine are contradictory. Further studies with large numbers of patients are needed to determine whether levobupivacaine and hyperbaric bupivacaine can be equal to bupivacaine in efficacy.

## Data Availability

The datasets used and/or analysed during the current study available from the corresponding author on reasonable request.

## Abbreviations

SA: Spinal anesthesia
SPO_2_: Saturation
ECG: Electrocardiogram
HR: Heart rate
CI: Confidence interval
SD: Standard deviation
nBP: Non-invasive blood pressure
VAS: Visual analog scale
